# A single-chain variable fragment intrabody prevents intracellular polymerization of Z *α*_1_-antitrypsin while allowing its antiproteinase activity

**DOI:** 10.1096/fj.14-267351

**Published:** 2015-03-10

**Authors:** Adriana Ordóñez, Juan Pérez, Lu Tan, Jennifer A. Dickens, Neda Motamedi-Shad, James A. Irving, Imran Haq, Ugo Ekeowa, Stefan J. Marciniak, Elena Miranda, David A. Lomas

**Affiliations:** *Department of Medicine, University of Cambridge, Cambridge Institute for Medical Research, Cambridge, United Kingdom; ^†^Department of Cell Biology, Genetics and Physiology, University of Malaga, Malaga, Spain; ^‡^Wolfson Institute for Biomedical Research, University College London, London, United Kingdom; and ^§^Department of Biology and Biotechnologies, “Charles Darwin,” and Pasteur Institute, Cenci Bolognetti Foundation, Sapienza University, Rome, Italy

**Keywords:** monoclonal antibody, scFv intrabody, serpin polymer, liver disease

## Abstract

Mutant Z *α*_1_-antitrypsin (E342K) accumulates as polymers within the endoplasmic reticulum (ER) of hepatocytes predisposing to liver disease, whereas low levels of circulating Z *α*_1_-antitrypsin lead to emphysema by loss of inhibition of neutrophil elastase. The ideal therapy should prevent polymer formation while preserving inhibitory activity. Here we used mAb technology to identify interactors with Z *α*_1_-antitrypsin that comply with both requirements. We report the generation of an mAb (4B12) that blocked *α*_1_-antitrypsin polymerization *in vitro* at a 1:1 molar ratio, causing a small increase of the stoichiometry of inhibition for neutrophil elastase. A single-chain variable fragment (scFv) intrabody was generated based on the sequence of mAb4B12. The expression of scFv4B12 within the ER (scFv4B12_KDEL_) and along the secretory pathway (scFv4B12) reduced the intracellular polymerization of Z *α*_1_-antitrypsin by 60%. The scFv4B12 intrabody also increased the secretion of Z *α*_1_-antitrypsin that retained inhibitory activity against neutrophil elastase. MAb4B12 recognized a discontinuous epitope probably located in the region of helices A/C/G/H/I and seems to act by altering protein dynamics rather than binding preferentially to the native state. This novel approach could reveal new target sites for small-molecule intervention that may block the transition to aberrant polymers without compromising the inhibitory activity of Z *α*_1_-antitrypsin.—Ordóñez, A., Pérez, J., Tan, L., Dickens, J. A., Motamedi-Shad, N., Irving, J. A., Haq, I., Ekeowa, U., Marciniak, S. J., Miranda, E., Lomas, D. A. A single-chain variable fragment intrabody prevents intracellular polymerization of Z *α*_1_-antitrypsin while allowing its antiproteinase activity.

*α*_1_-Antitrypsin deficiency is characterized by the accumulation of ordered polymers of mutant *α*_1_-antitrypsin within the endoplasmic reticulum (ER) of hepatocytes (1). The resulting inclusions cause a toxic gain of function that is associated with liver damage, whereas the plasma deficiency predisposes to early-onset lung emphysema caused by the loss of protease inhibition for several serine proteases, particularly neutrophil elastase (2, 3). The most common cause of *α*_1_-antitrypsin deficiency is homozygosity for the Z allele (E342K), which results in almost 90% of the synthesized *α*_1_-antitrypsin being degraded or retained as polymers within the ER of hepatocytes (1). Preventing the polymerization of Z *α*_1_-antitrypsin is an important therapeutic goal, but strategies that achieve this while abolishing its inhibitory activity would exacerbate lung disease in individuals with *α*_1_-antitrypsin deficiency. *α*_1_-Antitrypsin, as other members of the serine proteinase inhibitor (serpin) superfamily, exerts its antiproteinase activity by a unique mechanism (4, 5) involving a mobile and exposed reactive center loop (RCL). Binding of the target proteinase to the serpin (*e.g.,* neutrophil elastase to *α*_1_-antitrypsin) cleaves the serpin at a precise position within the RCL. As a result, the RCL inserts into *β*-sheet A becoming strand 4A, and the proteinase translocates to the lower pole of the 1:1 enzyme:inhibitor complex. Critically, insertion of the RCL into *β*-sheet A is also necessary for serpin polymerization (1, 6, 7).

The last decade has seen the development of different strategies to prevent the polymerization of Z *α*_1_-antitrypsin, aimed to therapeutic interventions. Three main approaches have been investigated: chemical chaperones (8–10), peptide analogs of the RCL (11–13), and small compounds to prevent the conformational transition that underlies polymer formation (14). Several *in vitro* studies have shown that peptide analogs of the RCL are able to block the *in vitro* polymerization of Z *α*_1_-antitrypsin but result in the loss of inhibitory activity (12, 13), whereas others have evaluated the effects of chemical chaperones or small molecules on Z *α*_1_-antitrypsin secretion in cell models or *in vivo*, without evidence of the functional activity of the secreted protein (8, 14). Finding a molecule that binds to *α*_1_-antitrypsin and prevents polymer formation while retaining antiproteinase functionality has thus become an important aim of the field.

Over the last few years, intracellularly expressed antibody fragments (intrabodies) have emerged as a powerful approach to modulate the function of targets in different intracellular compartments (15). The most common intrabody structure is the single-chain variable fragment (scFv), composed of 1 heavy and 1 light variable domains (V_H_ and V_L_, respectively) linked by the (Gly_4_Ser)_3_ synthetic flexible peptide. The scFv is the smallest fragment of an antibody capable of maintaining the antigen-binding specificity, with excellent properties of solubility, stability, and expression in mammalian cells (16). They can be targeted to subcellular compartments by incorporating trafficking signals specific for the ER, cytosol, nucleus, lysosomes, or mitochondria (17). Intrabodies have been used as research and therapeutic agents for a variety of protein conformational pathologies such as Alzheimer’s, Parkinson’s, and Huntington’s diseases and the prion encephalopathies (18), which share the feature of protein misfolding and accumulation with *α*_1_-antitrypsin deficiency and other serpinopathies (2).

Here we decided to take advantage of mAb technology to identify an interaction with Z *α*_1_-antitrypsin in which a ligand blocks polymer formation without compromising inhibitory activity. We report the production of a novel anti-Z *α*_1_-antitrypsin mAb, 4B12, able to block the polymerization of *α*_1_-antitrypsin *in vitro* while allowing the protein to retain inhibitory activity. Furthermore, we present the development of the scFv4B12 intrabody that greatly reduced the transition of monomeric Z *α*_1_-antitrypsin to pathologic polymers within the crowded environment of the ER in a cell model of disease. This intrabody increased the secretion of Z *α*_1_-antitrypsin while allowing its proteinase inhibitor activity. As the epitope is discontinuous and present in native, loop-inserted, and polymeric forms of the protein, the intrabody most likely acts by reducing the conformational dynamics of the protein rather than preferentially stabilizing one conformation over another.

## MATERIALS AND METHODS

### Mouse immunization, production of monoclonal antibodies, and ELISA assays

BALB/C mice were immunized with monomeric Z *α*_1_-antitrypsin purified from the plasma of a PI*ZZ homozygote, and the production of hybridoma was carried out as described previously (19). Hybridoma clones were first screened against purified monomeric Z *α*_1_-antitrypsin and then for their ability to block Z *α*_1_-antitrypsin polymerization. Selected hybridoma cells were subcloned by limiting dilution and expanded as cell lines. The resulting antibodies were characterized by ELISA assays as described previously (20). Briefly for (1) antigen-mediated ELISA, plates were coated with purified antigen proteins (monomer and polymer Z *α*_1_-antitrypsin) at 4 *µ*g/ml, followed by incubation with hybridoma culture media and with a rabbit anti-mouse horseradish peroxidase (HRP; Sigma-Aldrich, Dorset, United Kingdom); for (2) 2C1-antigen-9C5-HRP sandwich ELISA, plates were coated with our mouse monoclonal antibody specific for *α*_1_-antitrypsin polymers (mAb2C1) (20) at 2 *µ*g/ml, followed by incubation with unknown samples and with mAb 9C5-HRP (0.2 *µ*g/ml) (20); for (3) competitive ELISA, plates were coated with the antigen protein at 4 *µ*g/ml and mAbs to be tested were serially diluted followed by incubation with mAb 9C5-HRP (0.2 *µ*g/ml); and (4) *α*_1_-antitrypsin sandwich ELISA was performed as previously described (19, 20) using either our mAb3C11 (for total *α*_1_-antitrypsin) or mAb2C1 (for *α*_1_-antitrypsin polymers) as detection antibodies.

### Purification of mAb and Fab production

The full-length antibody was purified from hybridoma cell supernatant by using HiTrap Protein G Sepharose columns (GE Healthcare Life Sciences, Waukesha, WI, USA). The Fab fragment was produced using the Pierce Mouse IgG1 Fab and F(ab′)2 Preparation Kit (Life Technologies, Rockford, IL, USA).

### Inhibitory activity of *α*1-antitrypsin

The stoichiometry of inhibition of M (wild-type) or Z variants of *α*1-antitrypsin with human neutrophil elastase (HNE; Millipore, Billerica, MA, USA) was determined as described previously (21) in the presence/absence of an 8-fold molar excess of mAb4B12 (preincubated for 10 minutes) in 50 mM Tris, 0.5 M NaCl, and 0.25 M sucrose, pH 7.4, and with 500 *µ*M substrate. All values were normalized to the M *α*_1_-antitrypsin control.

### Formation of complexes with human neutrophil elastase

Complex formation with HNE (Calbiochem, Darmstadt, Germany) was evaluated in fetal bovine serum-free culture medium. Twenty microliters of culture medium was incubated for 45 minutes at 37°C with 10 ng HNE dissolved in 50 mM Tris buffer, pH 8.00. Adding 5 *µ*l 2× SDS loading buffer stopped the reaction, and samples were analyzed by SDS-PAGE and immunoblotted for *α*_1_-antitrypsin.

### Determination of the scFv sequences and construction of pCMV/scFv/myc/ER mammalian expression vectors

The scFv9C5 and scFv4B12 constructs were produced from mouse mAbs (both IgG1 *κ* isotype) against Z *α*_1_-antitrypsin. The sequences for V_H_ and V_L_ were determined as previously described (22). Primers to amplify the V_H_ and V_L_ regions were selected from the framework 1 region for V_H_ and V_L_ for the 5′ end and the constant regions for the 3′ end (23). Isolated heavy chains and light chains were cloned into pCR2.1 vector (Promega, Madison, WI, USA), and multiple clones were sequenced to identify the unique V_H_ and V_L_ sequences. Purified PCR products for the V_H_ and V_L_ were then linked with a flexible linker (Gly_4_Ser)_3_ by a 2-step overlapping PCR to produce the assembled scFv fragment. scFv4B12 was generated as an *in silico* sequence (with the same Gly-Ser linker and restriction sites) and purchased as a synthetic cDNA cloned into the pJ204 housekeeping plasmid (DNA2.0, Menlo Park, CA, USA). Both scFv9C5 and scFv4B12 were ligated into the cytomegalovirus promoter (pCMV)/myc/ER plasmid (pShooter; Invitrogen, Life Technologies, Carlsbad, CA, USA). The resulting scFv intrabodies contained the ER signal peptide, V_H_, interchain linker, V_L_, myc-tag, and the ER retention (KDEL) sequences. pCMV/scFv4B12/myc/ER (scFv4B12_KDEL_) was used as template to generate the pCMV/scFv4B12/myc (scFv4B12) without the ER retention sequence by site-directed mutagenesis (Agilent Technologies; Stratagen, La Jolla, CA, USA). The final constructs were confirmed by DNA sequencing. A full list of oligonucleotides used for cloning is included in **Table 1**.

**TABLE 1.** Oligonucleotides primers used for cloning in this study

Targeted sequence	Primer sequence	
	Forward	Reverse
Heavy chain (9C5/4B12)	5′ CTTCCGGAATTCSARGTNMAGCTGSAGSAGTC 3′	5′GGAAGATCTATAGACAGATGGGGTGTCGTTTTGGC 3′
Light chain (9C5/4B12)	5′ GGGAGCTCGAYATTGTGMTSACMCARWCTMCA 3′	5′ GGTGCATGCGATACAGTTGGTGCAGCATC 3′
V_H_9C5	5′ CAACTGCAGCTCGAGCAGGTGCAGCTGCAGCAGTCAGGGG 3′	5′ CTGAGGAGACGGTGACTGAGGTTCCTT 3′
V_L_9C5	5′ CGATATTGTGATCACCCAGACTCCAAA 3′	5′ TTTGATGCGGCCGCGTTTCAGCTCCAGCTTGGTCCCAG 3′

The same primer pairs were selected for PCR amplification of the mouse immunoglobulin heavy and light chains genes for both 9C5 and 4B12 mAbs. Highly degenerate primers were used for 5′ primers (S = G/C, R = A/G, N = A/C/G/T, M = A/C, and W = A/T). Specific primers for the amplification of the variable domains of the heavy and light chain (V_H_ and V_L_) for mAb9C5 are also shown. The underlined letters represent the cloning sites *Xho*I (ctcgag) and *Not*I (gcggccgc).

### COS-7 cell culture, intrabody transfections, and analysis

COS-7 cells were maintained as previously described (24) and cotransfected with 1.5 *µ*g of pcDNA3.1(+)-Z *α*_1_-antitrypsin and 2.5 *µ*g of scFv intrabodies using Lipofectamine 2000 (Invitrogen, Life Technologies). Cell lysates, SDS- and nondenaturing PAGE, immunoblotting, metabolic labeling, and immunoprecipitation and immunofluorescence analysis were performed as detailed previously (25). Antibodies and reagents used were as follows: anti-*α*_1_-antitrypsin monoclonal antibody (Abcam, Cambridge, United Kingdom), anti-myc-tag polyclonal antibody (Abcam), anti-KDEL antibody (Cell Signaling Technology, Danvers, MA, USA), anti- glyceraldehyde 3-phosphate dehydrogenase polyclonal antibody (Cell Signaling Technology), bafilomycin A1 (Sigma-Aldrich), an inhibitor of the V-ATPase essential for autophagosome maturation, and lactacystin (Calbiochem, San Diego, CA, USA), an irreversible proteasome inhibitor.

### Structural comparison

mAb4B12 recognizes an epitope common to native (Protein Data Bank ID code 1QLP), cleaved (Protein Data Bank ID code 1EZX), and latent (Protein Data Bank ID code 1IZ2) conformations, with candidate regions identified as follows: (1) surface-exposed residues rn (solvent-accessible surface area > 10 Å^2^) were identified using DSSP (26); (2) for the C*α* atom of each r_n_, an epitope-sized patch P_n_ of all surface residues with ≥1 side-chain atom within an 8 Å radius was identified; (3) for each patch P_n_, a least-squares superposition was performed between structures in pairs (S_i,j_) using LSQKAB (27) and the root mean square deviation (RMSD) between backbone atoms RMSD(P_n_,S_i,j_) calculated (deviations > 4.8 Å were treated as 4.8 Å). Any P_n_ with <3 amino acids, more than half of the positions displaced by >4.8Å, or overlapping with a known glycosylation site was noted; (4) for each pair S_i,j_ of structures, RMSD(Pr,Si,j) values were normalized giving 0 ≤ nRMSD(P_n_,S_i,j_) ≤ 1; and (5) the final value for each patch P_n_ was reported at central residue r as the average nRMSD(P_n_,S_i,j_).

### Statistical analysis

Statistical analysis by ANOVA, with a Bonferroni *post hoc* test or Student *t* test where appropriate, was performed using the GraphPad Prism program (GraphPad Software, La Jolla, CA, USA). Statistically significant changes (*P* < 0.05) are indicated.

## RESULTS

### Development of mAbs that interfere with the polymerization of Z *α*_1_-antitrypsin

**Figure 1.**
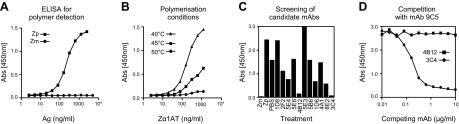
Production and characterization of polymerization-blocking mAbs to Z *α*_1_-antitrypsin. *A*) Typical curves obtained with purified Z *α*_1_-antitrypsin monomer (Zm) and polymer (Zp; 0.2 mg/ml at 60°C in PBS, pH 7.4, for 3 hours) in a sandwich ELISA (2C1-Ag-9C5-HRP). MAb2C1 recognizes only polymers and mAb9C5 recognizes all conformers of *α*_1_-antitrypsin. *B*) Conditions tested to establish an assay for identification of mAbs able to block heat-induced polymerization. *C*) Purified mAbs (50 *μ*g/ml) assessed in the polymerization assay shown in *B*. *D*) Competitive ELISA against the detection antibody (mAb9C5-HRP).

Three cell fusions were performed as described previously (19), and the resulting hybridoma cells (∼2500 wells) were screened for the presence of antibodies against Z *α*_1_-antitrypsin by antigen-mediated ELISA. Seventy positive wells were subjected to a secondary screening for mAbs able to modify the polymerization of Z *α*_1_-antitrypsin. Polymer formation was quantified by sandwich ELISA using our 2C1 polymer-specific mAb (20) (**Fig. 1*A***). The ability of candidate mAbs to block heat-induced polymerization was assessed by incubating Z *α*_1_-antitrypsin at concentrations similar to that expected for antibodies in a hybridoma supernatant (10–200 *µ*g/ml) and temperatures that could drive polymerization while allowing antibody-antigen interaction (40–50°C). The final assay was performed using 20 *µ*g/ml Z *α*_1_-antitrypsin, heated at 45°C for 45 hours (Fig. 1*B*). The fetal bovine serum contained in the media supernatant interfered with our assay by reducing polymerization (data not shown), and therefore each candidate antibody was purified and tested as a pure IgG reagent (Fig. 1*C*). Most mAbs did not modify Z *α*_1_-antitrypsin polymerization to any relevant extent. One of them caused an increase in polymer signal (5E3), 3 of them caused a mild decrease in polymer signal (5E4, 1D6, and 8E2), and 2 of them caused a strong decrease in polymer signal (4B12 and 3C4). These 2 antibodies were assessed in a competitive ELISA against mAb9C5-HRP to ensure that the reduction in signal was not caused by interference of the candidate mAb with the detection antibody. MAb3C4 competed the binding of 9C5-HRP to preformed Z *α*_1_-antitrypsin polymers (Fig. 1*D*), so it was discarded from our studies, whereas mAb4B12 showed no interference with polymer detection.

### Full-length and Fab region of mAb4B12 block the polymerization of Z *α*_1_-antitrypsin *in vitro*

**Figure 2.**
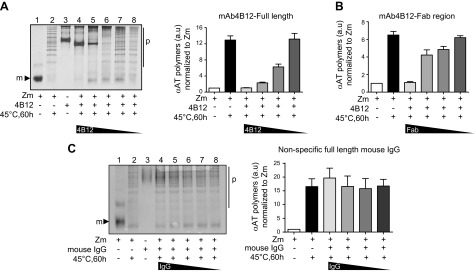
Polymerization blocking activity of the 4B12 monoclonal antibody. *A*) Nondenaturing PAGE followed by silver staining of 0.02 mg/ml monomeric Z *α*_1_-antitrypsin incubated with serial 1:2 dilutions of purified mAb4B12 starting from 1:1 molar ratio (0.063 mg/ml) at 45°C for 60 hours. Right) ELISA quantification of *α*_1_-antitrypsin polymers by sandwich ELISA (2C1-Ag-9C5-HRP) as means ± sem, *n* = 5, each performed in triplicate. *B*) Quantification of *α*_1_-antitrypsin polymers after incubation with purified Fab domain of mAb4B12 in the same conditions as *A* as means ± sem; *n* = 5, each performed in triplicate. *C*) Nondenaturing PAGE followed by silver staining of samples processed as in *A* but in the presence of nonspecific IgG_1_. Right) ELISA quantification for *α*_1_-antitrypsin polymers (*n* = 3). m, monomer; p, polymers.

The ability of the purified full-length 4B12 IgG to block polymerization was further assessed by nondenaturing PAGE (**Fig. 2*A***). mAb4B12 completely blocked heat-induced polymerization of Z *α*_1_-antitrypsin at a 1:1 molar ratio *in vitro* (lane 4), and the effect gradually decreased (polymerization increased, lanes 5–8) when the concentration of mAb4B12 was reduced. A specific 2C1 sandwich ELISA was used to quantify this effect with the same results (Fig. 2*A*, right). Similar data were obtained with the 4B12 Fab region (antigen-binding fragment), particularly at 1:1 molar ratio (Fig. 2*B*). An isotype-matching mouse IgG control (IgG_1_) had no effects on polymerization in the same conditions (Fig. 2*C*), supporting the specific blocking effect of mAb4B12. These results demonstrate that mAb4B12 is able to bind Z *α*_1_-antitrypsin and block its heat-induced polymerization at a 1:1 molar ratio *in vitro*.

### Identification of the scFv sequences for mAbs 4B12 and 9C5 and construction of intrabodies

**Figure 3.**
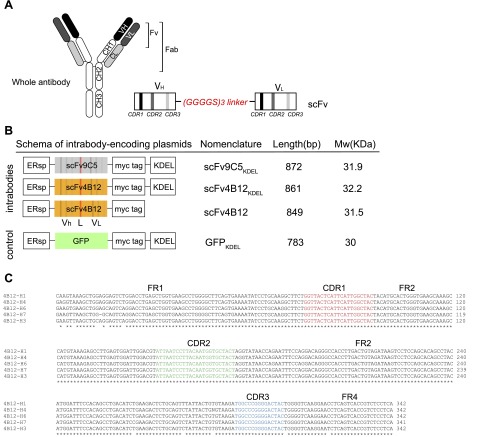
Construction of the scFv9C5 and scFv4B12 intrabodies. *A*) Representation of a whole antibody. Antigen specificity is defined by the Fab, composed of one constant (C) and one variable (V) domain of each of the heavy (H) and light (L) chains. The shortest variable-region fragment is called Fv. Schema of a single-chain variable fragment (scFv). CDR1–3 denotes complementarity-determining regions. *B*) Schema of the intrabody-encoding plasmids: 2 based on the mAb9C5 or mAb4B12 sequences with a myc epitope tag to facilitate detection and an ER retention signal (KDEL) (scFv9C5_KDEL_ and scFv4B12_KDEL_), and a third construct based on mAb4B12 but without the ER retention signal (scFv4B12). The GFP_KDEL_ vector expressed a nonrelated protein targeted to the ER. ERsp denotes ER signal peptide. *C*) Representative alignment of the DNA sequences of several clones for identifying the light chain sequence of mAb4B12. The frameworks regions (FR) and complementary regions (CDR) are indicated.

The striking polymer-blocking properties of mAb4B12 *in vitro* encouraged us to evaluate the effect of this antibody in a cell model of disease. To this end, we generated the scFv, composed of the V_H_ and V_L_ domains joined by a flexible linker (Gly_4_Ser)_3_ (**Fig. 3*A, B***). Two hybridoma cell lines were used as the source of RNA for scFv design: cells producing mAb4B12 and mAb9C5, an antibody against Z *α*_1_-antitrypsin polymers that recognizes all conformers of *α*_1_-antitrypsin (20) but that does not block polymer formation, used here as a negative control. The unique cDNA sequence for the variable domains (V_H_ and V_L_) containing the complementarity-determining regions (hypervariable domains) responsible for antigen binding were identified by comparison of multiple sequenced clones to the mouse IG set from the ImMunoGeneTics information system for V-QUEry and STandardization (IMGT/V-QUEST) reference directory (28) (Fig. 3*C*). The resulting scFv4B12 and scFv9C5 constructs were sequenced, revealing a 750 bp open reading frame full-length cDNA, encoding a 244 amino acid protein with an estimated molecular weight of 27 kDa (Supplemental Fig. S1).

The Z (E342K) variant of *α*_1_-antitrypsin forms ordered polymers and intracellular inclusions that result in gross changes in the luminal environment of the ER (25). To test the efficacy of the scFv in this pathologically relevant context, the scFv-encoding sequences were cloned into an ER-targeting pCMV/ER mammalian expression vector, which contained the ER signal peptide and a KDEL-ER retention sequence. The 3 intrabodies generated are shown in Fig. 3*B*. Two were based on mAb9C5 (scFv9C5_KDEL_) and mAb4B12 (scFv4B12_KDEL_), containing the KDEL sequence. Previous reports have shown that intrabodies retained within the ER by the KDEL sequence are not secreted (29) and act as intracellular anchors preventing the secretion of the target proteins (30). Therefore, we also generated an intrabody based on mAb4B12 without the KDEL sequence (scFv4B12), which should allow normal trafficking of *α*_1_-antitrypsin. The myc epitope and the ER retention signal added ∼5 kDa, so the expected size of the intrabodies was approximately 32 kDa. A green fluorescent protein (GFP)_KDEL_ vector (Invitrogen, Carlsbad, CA, USA) was used as an additional negative control.

### Efficient expression of intrabodies within the secretory pathway

**Figure 4.**
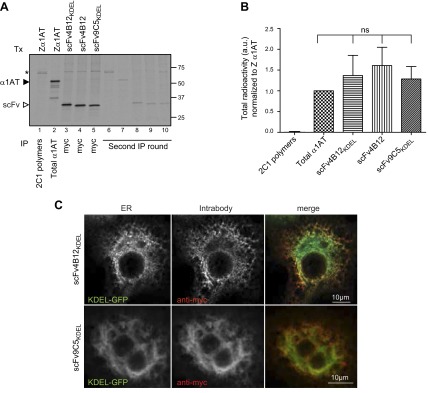
Intrabodies are efficiently expressed within the ER of COS-7 cells. *A*) Cells were transfected (Tx) with Z *α*_1_-antitrypsin or each intrabody: scFv4B12_KDEL_, scFv4B12, or scFv9C5_KDEL_ for 24 hours and pulse-labeled with [^35^S]-Met/Cys for 20 minutes. Total *α*_1_-antitrypsin and intrabody proteins were immunoprecipitated from the cell lysates, separated by SDS-PAGE, and subjected to autoradiography. MAb2C1 was also used to quantify polymer formation. The asterisk indicates a nonspecific band. *B*) Protein expression levels represented as means ± sem; Mann-Whitney test, *n* = 3; ns, nonsignificant. *C*) Confocal microscopy of fixed cells transfected with scFv4B12_KDEL_ or scFv9C5_KDEL_ in combination with the KDEL-GFP plasmid as an ER marker and stained with an anti-myc antibody to visualize the intrabodies.

We have previously shown that polymers of Z *α*_1_-antitrypsin accumulate within the ER of COS-7 cells, reproducing the secretory defects seen in patients (20). Hence, we decided to use this model system to evaluate the correct expression of the different intrabodies. COS-7 cells were transfected with Z *α*_1_-antitrypsin or each intrabody and after 24 hours were pulse-labeled with [^35^S]-Met/Cyst. Protein expression levels were determined by immunoprecipitation with either an antibody against all conformers of *α*_1_-antitrypsin or against the myc-tag for the intrabodies (**Fig. 4*A***). As expected, a 52 kDa band was detected for *α*_1_-antitrypsin (lane 2), and bands of ∼32 kDa were detected in cells expressing scFv4B12_KDEL_, scFv4B12, and scFv9C5_KDEL_ (lanes 3–5). The slightly faster migration of scFv4B12 and scFv9C5_KDEL_ compared with scFv4B12_KDEL_ (Fig. 4*A*; lanes 4 and 5 *vs*. lane 3) was in agreement with their predicted molecular weights. To ensure correct quantification, cell lysates were subjected to a second round of immunoprecipitation. There were similar expression levels for all proteins when total radioactivity was corrected for the number of Cys/Met residues contained in Z *α*_1_-antitrypsin (13 Met/Cys) and in the intrabodies (all of them with 9 Met/Cys; Fig. 4*B*). As a control, we evaluated the presence of polymers in the cell lysates; no polymers were detected at the short time of the pulse (lane 1), as reported previously for polymerogenic mutants of the neuronal serpin neuroserpin (31). Immunofluorescence staining revealed a reticular distribution pattern for the intrabodies that colocalized with the ER as detected with an anti-KDEL antibody (Fig. 4*C*). These results demonstrate that all the intrabodies were efficiently expressed in COS-7 cells at protein levels comparable to *α*_1_-antitrypsin and that they were successfully driven into the ER.

### scFv4B12 intrabodies block intracellular polymerization of Z *α*_1_-antitrypsin

**Figure 5.**
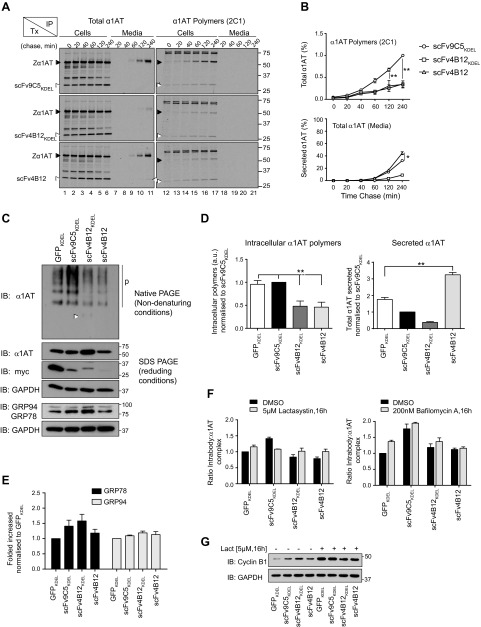
The scFv4B12 intrabody reduces the polymerization of Z *α*_1_-antitrypsin in a cell model of disease. *A*) COS-7 cells were cotransfected (Tx) with Z *α*_1_-antitrypsin and scFv9C5_KDEL_, scFv4B12_KDEL_, or scFv4B12. Twenty-four hours after transfection, cells were pulse-labeled with [^35^S]-Met/Cys for 20 minutes and chased for the indicated times. *α*_1_-Antitrypsin from cell lysates and culture media was immunoprecipitated with a polyclonal antibody (total *α*_1_-antitrypsin) or mAb2C1 (*α*_1_-antitrypsin polymers) by splitting each sample into 2 equal aliquots. Samples were resolved by 10% (v/v) SDS-PAGE and detected by autoradiography. Black arrowheads indicate Z *α*_1_-antitrypsin (intracellular 52 kDa species and extracellular 55 kDa species) and white arrowheads indicate scFvs (32 kDa species). *B*) Quantification graphs from the experiment performed in *A* (*n* = 3); *P < 0.05; **P < 0.01, according to analysis of variance test, followed by Bonferroni’s *post hoc* test. *C*) COS-7 cells were cotransfected with Z *α*_1_-antitrypsin and scFv9C5_KDEL_, scFv4B12_KDEL_, scFv4B12, or GFP_KDEL_ as indicated. Cell lysates were collected after 24 hours and analyzed either on 10% (v/v) SDS- or nondenaturing PAGE and immunoblotted for *α*_1_-antitrypsin, myc-tag (scFv), KDEL (for detection of both GRP78 and GRP94 ER chaperones), and glyceraldehyde 3-phosphate dehydrogenase (GAPDH). *D*) Cell lysates from experiments performed in *C* were subjected to sandwich ELISA for quantification of intracellular *α*_1_-antitrypsin polymers (using mAb2C1; right), and both cell lysates and culture media were analyzed by sandwich ELISA to quantify the percentage of secreted total *α*_1_-antitrypsin (using mAb3C11, which recognizes all conformers of *α*_1_-antitrypsin and does not compete with mAb9C5; left). Histograms represent the means ± sem of 5 independent experiments. **P < 0.01; Mann-Whitney test. *E*) Histogram of 3 independent experiments as in *C* showing fold increase of GRP78 and GRP94 normalized to loading control and then to GFP_KDEL_ (means ± sem). Mann-Whitney test (*n* = 3) showed nonsignificant differences. *F*) COS-7 cells cotransfected as in *C* were treated either with 5 *µ*M lactacystin (left) or 200 nM bafilomycin (right) for 16 hours. *α*_1_-Antitrypsin:intrabody complex was quantified by sandwich ELISA using a polyclonal anti-*α*_1_-antitrypsin antibody for capture and an anti-myc antibody for detection. Graphs represent the means ± sd of 2 or 3 independent experiments. *G*) Cell lysates from *F* (left) were immunoblotted for cyclin B1, a rapidly degraded proteasomal substrate, as a positive control for blocking of proteasomal activity with lactacystin.

We next assessed the ability of the scFv4B12 intrabodies to prevent the polymerization of Z *α*_1_-antitrypsin in our cell model of disease by performing metabolic labeling of cells transiently cotransfected with Z *α*_1_-antitrypsin and the intrabodies at a 1:2 molar ratio. The scFv4B12 was expressed within the ER (scFv4B12_KDEL_) and along the secretory pathway (scFv4B12), and we used the scFv9C5_KDEL_, which was also able to bind to Z *α*_1_-antitrypsin but not block polymerization, as a negative control. Cells were incubated with [^35^S]-Met/Cys and chased for up to 4 hours (**Fig. 5*A***). Immunoprecipitation of *α*_1_-antitrypsin polymers from cell lysates with mAb2C1 confirmed that the scFv4B12_KDEL_ and scFv4B12 intrabodies reduced polymer formation by up to 60% (Fig. 5*A*; lanes 12–17 black arrowhead, and Fig. 5*B*, upper), without any apparent effect on the translation of total *α*_1_-antitrypsin (Fig. 5*A*, lane 1). In cells transfected with scFv4B12 intrabody, reduced polymerization was correlated with secretion levels slightly higher than those observed in control cells transfected with scFv9C5_KDEL_ (Fig. 5*B*, lower); in contrast, cells expressing the scFv4B12_KDEL_ intrabody showed reduced levels of *α*_1_-antitrypsin secretion (Fig. 5*A*, lanes 7–11, and Fig. 5*B*, lower). All intrabody proteins efficiently bound Z *α*_1_-antitrypsin, as indicated by the 32 kDa bands coimmunoprecipitated from the cell lysates (Fig. 5*A*, lanes 1–6, white arrowheads). The scFv4B12 could also be faintly detected in the cell media (Fig. 5*A*, lanes 10–11, white arrowhead). Interestingly, the intrabodies showed capacity to bind both monomeric (Fig. 5*A*, lanes 1–6, white arrowheads) and polymeric (Fig. 5*A*, lanes 12–17, white arrowheads) Z *α*_1_-antitrypsin. Taken together, these data demonstrate that expression of the scFv region of the mAb4B12 as an intrabody, whether or not fused to an ER retention signal, reduced the intracellular polymerization of Z *α*_1_-antitrypsin in a cell model of disease.

Next we evaluated the effects of the scFv4B12_KDEL_ and scFv4B12 intrabodies at longer time points (24 hours) by steady-state analysis of COS-7 cells transiently cotransfected in the same conditions used above for pulse-chase experiments and introducing an additional GFP_KDEL_ negative control. When assessed by nondenaturing and SDS-PAGE, cells expressing either scFv4B12_KDEL_ or scFv4B12 showed a clear reduction in intracellular polymers (Fig. 5*C*, upper), and for scFv4B12_KDEL_, a faint monomer band could be seen in the cells lysates (white arrowhead). The scFv proteins could be visualized using an anti-myc-tag antibody (Fig. 5*C*, myc panel). Quantification by ELISA using the mAb2C1 polymer-specific antibody confirmed that, compared with control cells, both the scFv4B12_KDEL_ and scFv4B12 intrabodies reduced the levels of intracellular Z *α*_1_-antitrypsin polymers by up to 60% (Fig. 5*D*, left). At steady state, the levels of total *α*_1_-antitrypsin secreted into the media were lower when cells expressed intrabodies to *α*_1_-antitrypsin containing the KDEL sequence (scFv9C5_KDEL_ and scFv4B12_KDEL_), whereas secretion from cells expressing scFv4B12 was significantly higher to that seen for cells transfected with a nonrelated protein (GFP_KDEL_; Fig. 5*D*, right). These results extend our initial observation of a small but significant increase in secretion in the presence of the scFv4B12 intrabody after 4 hours by pulse chase, suggesting that changes in secretion are better evaluated at longer times.

Because the expression of intrabodies with the KDEL sequence caused their retention within the ER, we assessed whether they could induce ER stress by looking at protein levels of 2 main ER luminal chaperones: BiP/GRP78 and GRP94. Our results showed a slight but not significant increase for both proteins (mainly for BiP/GRP78) on expression of scFv9C5_KDEL_ and scFv4B12_KDEL_ (Fig. 5*C*, GRP94/GRP78 panel, and Fig. 5*E*). In contrast, cells expressing scFv4B12, which allowed protein trafficking, showed levels of GRP78 and GRP94 similar to cells expressing the GFP_KDEL_ control. We also evaluated whether the intrabody-*α*_1_-antitrypsin complex was subjected to degradation by the proteosomal or autophagic pathways, but inhibiting these pathways with specific pharmacologic agents produced negative results (Fig. 5*F*). These results demonstrate that intracellular polymerization of Z *α*_1_-antitrypsin is efficiently prevented by scFv4B12, causing increased secretion in the absence of marked ER stress or activation of degradative pathways.

### Z *α*_1_-antitrypsin retains inhibitory activity *in vitro* when bound to the mAb4B12 and after secretion from cells coexpressing the scFv4B12 intrabody

**Figure 6.**
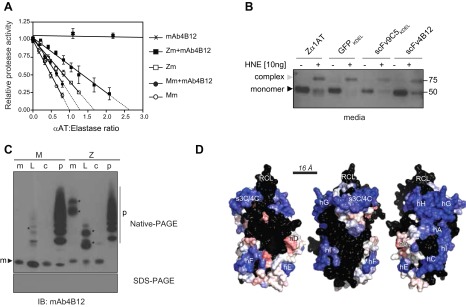
MAb4B12 recognizes a conformational epitope in which binding is compatible with preservation of the inhibitory activity of Z *α*_1_-antitrypsin. *A*) Neutrophil elastase inhibitory activity of monomeric M (Mm) and Z (Zm) *α*_1_-antitrypsin in the presence and absence of mAb4B12. Samples were preincubated with an excess of antibody and mixed at different ratios with HNE. Residual enzyme activity is shown as a proportion of the uninhibited control, and all intercept values are normalized to that of wild-type M *α*_1_-antitrypsin. The error bars are se for 3–4 independent experiments. *B*) Culture media supernatant from cells transfected as in Fig. 5*C* were incubated with HNE and resolved by SDS-PAGE followed by immunoblot for *α*_1_-antitrypsin. Gray arrowheads indicate the *α*_1_-antitrypsin:HNE complex and black arrowheads unbound *α*_1_-antitrypsin. All data are means ± sem. *C*) Representative immunoblot, using mAb4B12 as the primary antibody, shows recognition of the native monomer (m), latent (L), cleaved (c), and polymeric (p) forms of M and Z *α*_1_-antitrypsin when separated by nondenaturing PAGE (upper), but not after denaturing for SDS-PAGE (lower). The stronger bands indicated with asterisks represent short chain polymers. *D*) A rolling representation of the solvent-accessible surface of the native fold of *α*_1_-antitrypsin, with successive images rotated by 120°. Surface patches 16 Å in diameter (approximately the size of an antibody-antigen interface) were identified and classified, and their central residue was colored according to whether they were most (blue) or least (red) structurally conserved between structures of the native, cleaved, and latent conformations. For patches in which more than half of the constituent residues have been displaced by >4.8Å or for those that overlap a glycosylation-compatible asparagine residue, the central position has been colored black. Image prepared using Pymol (Schrödinger, Camberley, United Kingdom).

We next assessed the inhibitory activity of *α*_1_-antitrypsin when bound to mAb4B12. *In vitro* binding to mAb4B12 increased the stoichiometry of inhibition of Z *α*_1_-antitrypsin for neutrophil elastase from 1.7 ± 0.1 to 2.6 ± 0.2 (**Fig. 6*A*** and **Table 2**). Retention of inhibitory activity by antibody-bound *α*_1_-antitrypsin indicates that the mode of action of mAb4B12 is not simply steric interference with the RCL or *β*-sheet A; partial suppression of inhibitory activity is suggestive of an effect on the insertion mechanism itself. Our metabolic labeling experiments suggested that a small fraction of the total secreted Z *α*_1_-antitrypsin was in complex with scFv4B12 (Fig. 5*A*, lower, lane 11). The activity of this complex was investigated by assessing the formation of an SDS-stable complex with HNE (Fig. 6*B*). The results showed that Z *α*_1_-antitrypsin secreted from cells cotransfected with scFv4B12 was able to form an ∼75 kDa complex following the addition of 10 ng of HNE, in agreement with our *in vitro* experiments. This was similar to Z *α*_1_-antitrypsin secreted by cells expressing the control GFP_KDEL_ and scFv9C5_KDEL_. These experiments suggest that the coexpression of the scFv4B12 does not inactivate Z *α*_1_-antitrypsin as a proteinase inhibitor.

**TABLE 2.** The stoichiometry of inhibition of the interaction between α_1_-antitrypsin and HNE in the presence of mAb4B12

Sample	Stoichiometry of inhibition
Mm	1
Mm+mAB4B12	1.3 ± 0.1
Zm	1.7 ± 0.1
Zm+mAb4B12	2.6 ± 0.2

### Epitope recognized by mAb4B12 is discontinuous and most likely lies on the helix-rich face of *α*_1_-antitrypsin

To determine whether the epitope recognized by scFv4B12 is unique to the native conformation, *α*_1_-antitrypsin in different conformational states was resolved by nondenaturing PAGE and detected by immunoblot with mAb4B12 (Fig. 6*C*, upper). All conformations, monomeric and oligomeric, were recognized. However, on separation by SDS-PAGE, this reactivity was lost (Fig. 6*C*, lower), indicating that the epitope is discontinuous and therefore the species recognized in cells by scFv4B12 is not in an unfolded state. The conservation of the epitope across multiple conformations of *α*_1_-antitrypsin indicates that it must occur in a structurally invariant region. This observation was used as the basis of a comparative analysis of the crystal structures corresponding to the native, RCL cleaved, and latent forms. An iterative approach was used in which all possible patches of surface-accessible residues of 16 Å diameter in one structure were compared with the equivalent residues in another structure. The combined result was a surface map indicating the degree to which these patches were structurally conserved between different conformations (Fig. 6*D*). Consistent with the observation that binding to mAb4B12 remains compatible with protease inhibition, the RCL and much of the *β*-sheet A-dominated region flanking helix F can be excluded as the common binding site because of substantial differences between the structures (colored black in Fig. 6*D*). The helix-rich region spanning helices A, C, G, H, and I, combined with strands 3C and 4C, form the most extensive surface that is compatible with interaction with the antibody. Notably, the mild polymerogenic mutants R39C (I) and E264V (S), which disrupt stabilizing interactions linking secondary structural elements, occur within this region (32), in addition to latch mutations in *α*_1_-antitrypsin (33).

## DISCUSSION

Conformational diseases are characterized by protein misfolding and intra- or extracellular accumulation of pathologic protein aggregates (34). Most of these disorders still lack an effective treatment, in part because of the difficulty of targeting the subcellular compartments in which mutant proteins accumulate. Antibody-based strategies are emerging as potent therapies as the small size of scFv intrabodies facilitates their delivery to subcellular compartments, making them suitable as therapeutic agents and tools for the study of conformational disorders (35). Indeed, a variety of ER-targeted intrabodies, for example, against the *β*-amyloid peptide (Alzheimer’s disease) (36) and to the prion protein (prion disease) (37), have been shown to prevent protein aggregation by *in vitro* and *in vivo* studies. Although *α*_1_-antitrypsin deficiency represents one of the best models of conformational disease (38), intrabodies have not been investigated thus far as a strategy to alleviate Z *α*_1_-antitrypsin polymer overload within the ER of hepatocytes. The case of *α*_1_-antitrypsin deficiency is complicated by the simultaneous need to retain the inhibitory function of the protein to protect the lungs from excessive proteolysis. Here we sought to use the exploratory potential of mAb to identify a binding site that can block the polymerization of Z *α*_1_-antitrypsin without loss of inhibitory activity.

We report the development of the novel mAb4B12 that was identified by its ability to block heat-induced polymerization of Z *α*_1_-antitrypsin *in vitro.* The isolated Fab domain of this antibody showed similar properties, whereas a nonspecific IgG of the same isotype had no effect on polymer formation. To our knowledge, this is the first mAb (whole or Fab region) that robustly inhibits the polymerization of Z *α*_1_-antitrypsin. We thus generated 4B12-based and control intrabodies targeted to the ER by a C-terminal KDEL sequence. A version of the scFv4B12 intrabody was also prepared without the KDEL sequence to allow normal trafficking of *α*_1_-antitrypsin and prevent polymer formation in post-ER compartments, where lower pH (pH 5.5–4.8 in secretory vesicles) (39) could favor polymer formation as described *in vitro* at low pH (pH 5.5) (40). Our data show that the scFv4B12_KDEL_ and scFv4B12 intrabodies can inhibit the intracellular polymerization of Z *α*_1_-antitrypsin by up to 60%, suggesting that most of the polymerization occurs within the ER. Parallel experiments with a control intrabody (scFv9C5_KDEL_) that recognizes all conformers of *α*_1_-antitrypsin confirmed that the polymerization blocking effect was specific to the 4B12-based intrabodies. It is notable that an antibody identified by its ability to block heat-induced polymerization of native Z *α*_1_-antitrypsin *in vitro* also blocked polymer formation in a cell model of disease. This is in keeping with the data from the antipolymer mAb2C1, showing that heating Z *α*_1_-antitrypsin gives rise to an epitope on *α*_1_-antitrypsin polymers that is also formed *in vivo* (20). It also suggests that the majority of intracellular polymers form from “near-native” folded *α*_1_-antitrypsin. The failure to completely abolish intracellular polymerization may result from insufficient intrabody accessing the ER, the shielding of the blocking epitope in the crowded environment of the ER, or alternative polymers forming by pathways that are not blocked by binding to the scFv4B12 epitope.

To date, most studies that have attempted to block the polymerization of Z *α*_1_-antitrypsin have used chemical chaperones, synthetic peptides, and small compounds (8–14), some of them with promising results *in vitro*. Sugar and alcohol molecules can reduce the rate of *in vitro* polymerization by 2.9- to 7.7-fold (10), but the few studies that have evaluated the effect of chemical chaperones or small molecules in cell systems or *in vivo* have focused on Z *α*_1_-antitrypsin secretion, with no direct evidence of polymer reduction (8, 14). Here we show that the 4B12-based intrabodies were able to prevent polymer formation *in vitro* and to reduce it by up to 2.5-fold in a cell model of disease and that the trafficking-competent scFv4B12 intrabody leads to a significant improvement of Z *α*_1_-antitrypsin secretion. Furthermore, the scFv4B12 intrabody did not elicit ER stress, suggesting that this protein had no major secondary effects in the ER. Previous evidence has shown that scFv expressed in the secretory compartment can be subjected to proteasome degradation (41); however, in our hands the Z *α*_1_-antitrypsin-intrabody complex was not degraded by the proteasome or by autophagy, in agreement with the absence of marked ER stress. Future studies will assess the effects of the 4B12 intrabody in an *in vivo* model of Z *α*_1_-antitrypsin polymer formation.

Preventing Z *α*_1_-antitrypsin polymerization is an important therapeutic goal, given the link between polymer deposition and liver disease (42). However, strategies that achieve this by filling the “gap” between strands 3 and 5 of *β*-sheet A would compromise the inhibitory activity of *α*_1_-antitrypsin, which requires internalization of the cleaved RCL in the core of this *β*-sheet. This is an important issue, in particular with reactive loop peptidomimetics and the first generation of small molecule polymer blockers (1, 14). In contrast, Z *α*_1_-antitrypsin bound to mAb4B12 retained almost two thirds of its inhibitory activity against neutrophil elastase *in vitro*, supporting the use of mAb4B12 (as whole antibody or intrabody) as a research tool in *α*_1_-antitrypsin deficiency. This suggests a mechanism of action whereby mAb4B12 increases resistance of the variant to opening of its central *β*-sheet. However, it does not achieve this by simply preferentially stabilizing the 5-stranded native state, as the epitope is present across the conformational repertoire, including the 6-stranded form, of the protein. Instead, the antibody appears to act by reducing the structural dynamics of the antigen in a conformation-neutral fashion. It is therefore remarkable that this antibody prevents polymerization in the cellular context at all; this is highly suggestive of a mechanism whereby the antibody exploits a folding/polymerization pathway in which polymerization ensues from a substantially folded, native-like form.

Our results open up the use of intrabodies in the field of serpinopathies, with unique advantages over existing gene-targeted techniques such as silencing/interfering RNA or RNA aptamers (called “intramers” when intracellularly expressed) (43, 44). Particularly, intrabodies can target proteins in different cellular compartments; present very stable expression in mammalian cells compared with small siRNAs (45) or intramers (46); are highly specificity to the target; and can reroute the misfolding-prone protein from the accumulation site (36). However, as for other gene therapy strategies, the *in vivo* delivery of intrabodies is challenging. Delivery using adeno-associated virus is currently preferred for gene therapy, as a nonintegrative vector that can highly infect dividing cells and has recently shown promising results *in vivo* (47). Targeting Z *α*_1_-antitrypsin would also require a hepatotropic delivery system to ensure sufficient amounts of intrabody expressed in hepatocytes. Recent reports using vectors driven by humanized hepatocyte-specific promoters (such as the apolipoprotein locus control region and the human *α*_1_-antitrypsin promoter—encoding the apolipoprotein locus control region and the human *α*_1_-antitrypsin—or the albumin promoter) have successfully achieved sustained transgene expression in the liver *in vivo* (48), opening the way to gene therapy applications for liver disease.

This study provides the first step in the use of intrabodies or other molecules designed to mimic their binding properties as an approach to prevent the polymerization of Z *α*_1_-antitrypsin while preserving its inhibitory activity and encourages future research in their therapeutic application. In addition, further studies performed in more physiologic systems and evaluating the structural basis of the blocking properties of mAb4B12 may provide insights into the stability of *α*_1_-antitrypsin and the polymerization mechanism that underlies the ensuing conformational disease.

## Supplementary Material

Supplemental Data

## Abbreviations

ER: endoplasmic reticulum
GFP: green fluorescent protein
HNE: human neutrophil elastase
HRP: horseradish peroxidase
pCMV: cytomegalovirus promoter
RCL: reactive center loop
RMSD: root mean square deviation
scFv: single-chain variable fragment
V_H_: variable heavy chain
V_L_: variable light chain
