# Supplementary material for: A single-chain variable fragment intrabody prevents intracellular polymerization of Z α1-antitrypsin while allowing its antiproteinase activity
Source: FASEB J. 2015 Mar 10;29(6):2667–78. doi: 10.1096/fj.14-267351 (PMC4548814; doi:10.1096/fj.14-267351)
Supplement: Supplemental Data [file supp_29_6_2667__index.html]

A single-chain variable fragment intrabody prevents intracellular polymerization of Z α1-antitrypsin while allowing its antiproteinase activity — A single-chain variable fragment intrabody prevents intracellular polymerization of Z α1-antitrypsin while allowing its antiproteinase activity — Supplemental Data 

# A single-chain variable fragment intrabody prevents intracellular polymerization of Z *α*1-antitrypsin while allowing its antiproteinase activity

## Supplemental Data

**Files in this Data Supplement:**

- Supplemental Data
- Supplemental Data
