# Supplementary material for: A single-chain variable fragment intrabody prevents intracellular polymerization of Z α1-antitrypsin while allowing its antiproteinase activity
Source: FASEB J. 2015 Mar 10;29(6):2667–78. doi: 10.1096/fj.14-267351 (PMC4548814; doi:10.1096/fj.14-267351)
Supplement: Supplemental Data [file supp_fj.14-267351_Supplemental_Figure1.pdf]

(a)

ER signal peptide

ATG GGA TGG AGC TGT ATC ATC CTC TTC TTG GTA GCA ACA GCT ACA GGC GCG CAC TCC

Xho1 *Vh→*

CAG GTC CAA CTG CAG GTC GAC CTC GAG CAA GTC AAG CTG GAG GAG TCT GGA CCT GAG

CDR1h

CTG GTG AAG CCT GGG GCT TCA GTG AAA ATA TCC TGC AAG GCT TCT GGT TAC TCA TTC

ATT GGC TAC TAC ATG CAC TGG GTG AAG CAA AGC CAT GTA AAG AGC CTT GAG TGG ATT

CDR2h

GGA CGT ATT AAT CCT TAC AAT GGT GCT ACT AGG TAT AAC CAG AAT TTC CAG GAC AGG

GCC ACC TTG ACT GTA GAT AAG TCC TCC AGC ACA GCC TAC ATG GAT TTC CAC AGC CTG

CDR3h

ACA TCT GAA GAC TCT GCA GTC TAT TAC TGT GTA AGA TGG CCC GGG GAC TAC TGG GGT

CAA GGA ACC TCA GTC ACC GTC TCC TCA **GGT GGA GGC GGT TCA GGC GGA GGT GGC TCT**

VL→

GGC GGT GGC GGA TCG GAT ATT GTG ATG ACC CAG ACT CCA TCT TCA CTG TCT GCA TCT

CDR1L

CTG GGA GGC AAA GTC ACC ATC ACT TGC AAG GCA AGC CAA GAC ATT AAC AAC TAT ATA

GCT TGG TAC CAG CTC AAG CCT GGA AAA GGT CCT AGA CAA CTC ATA CAT TAC ACA TCT

AAA TTA CAG CCA GGC ATC CCA TCA CGG TTC AGT GGA AGT GGG TCT GGG AGT GAT TAT

CDR2L

TCC TTC AGC ATC AGC AAC CTG GAG CCT GAA GAT ATT GGA ACT TAT TAT TGT CTA CGC

CDR3L *Not1*

TAT GAG GAT CTG TGG ACG TTC GGT GGA GGC ACC AAG TTG GAA ATC AAA GCG GCC GCA

myc epitope *KDEL sequence*

GAA CAA AAA CTC ATC TCA GAA GAG GAT CTG AAT GGG GCC GCA AGC GAG AAG GAC GAG

CTG TAG

scFv4B12$_{KDEL}$

(b)

ER signal peptide

ATG GGA TGG AGC TGT ATC ATC CTC TTC TTG GTA GCA ACA GCT ACA GGC GCG CAC TCC

Xho1 *Vh →*

CAG GTC CAA CTG CAG GTC GAC CTC GAG CAG GTG CAG CTG CAG CAG TCA GGG GCA GAG

CDR1h

CTT GTG AAG CCA GGG GCC TCA GTC AAG TTG TCC TGC ACA GCT ACT GGC TTC AAC ATT

AAA GAC ACC TAT ATG CAC TGG GTA AAA CAG AGG CCT GAA CAG GGC CTG GAG TGG ATT

CDR2h

GGA AGG ATT GAT CCT GCG AAT GGT AAT ACT AAA TAT GAC CCG AAG TTC CAG GGC AAG

GCC ACT TTA ACA GCA GAC ACA TCC TCC AAC ACA GCC TAC CTG CAG CTC AGC AGC CTG

CDR3h

ACA TCT GAG GAC ACT GCC GTC TAT TAC TGT GCT AGA AAA AGG TAC TCT ATG GAC TAC

TGG GGT CAA GGA ACC TCA GTC ACC GTC TCC TCA **GGT GGA GGC GGT TCA GGC GGA GGT**

VL→

GGC TCT GGC GGT GGC GGA TCG CGA TAT TGT GAT CAC CCA GAC TCC AAA TTC CTG CTT

CDR1L

GTA TTA GCA GGA GAG AGG GTT ACC ATA ACC TGC AAG GCC AGT CAG AGT GTG AGT AAT

GAT GTA GGT TGG TAC CAA CAG AAG CCA GGG CAG CCT CCT AAA CTG CTG ATA TAC AAT

CDR2L

GCA TCC AAT CGC AAA AAT GGA GTC CCT GAT CGC TTC ACT GGC AGT GGA TAT GGG ACG

GAT TTC ACT TTC ACC ATC AGC ACT GTG CAG GCT GAA GAC CTG GCA GTG TAT TTC TGT

CDR3L

CAG CAG GAT CAT AGT TTT CCT CTC AAG TTC GGT GCT GGG ACC AAG CTG GAG CTG AAA

Not1 *myc epitope*

GCG GCC GCA GAA CAA AAA CTC ATC TCA GAA GAG GAT CTG AAT GGG GCC GCA AGC GAG

KDEL sequence

AAG GAC GAG CTG TAG

scFv9C5$_{KDEL}$
