# Supplementary material for: A single-chain variable fragment intrabody prevents intracellular polymerization of Z α1-antitrypsin while allowing its antiproteinase activity
Source: FASEB J. 2015 Mar 10;29(6):2667–78. doi: 10.1096/fj.14-267351 (PMC4548814; doi:10.1096/fj.14-267351)
Supplement: Supplemental Data [file supp_fj.14-267351_Supplemental_Data.doc]

**SUPPLEMENTAL DATA**

**FIGURE**

**Fig. S1. Construction of the scFv9C5 and scFv4B12 intrabodies.** (a) DNA sequence of the ER-targeted 4B12 intrabody against α_1_-antitrypsin (scFv4B12_KDEL_). **(b)** DNA sequence of the ER-targeted 9C5 intrabody against α_1_-antitrypsin (scFv9C5_KDEL_). The linker that joins the V_H_ and V_L_ domains is shown in red.
